# Environmental Air Pollutants Inhaled during Pregnancy Are Associated with Altered Cord Blood Immune Cell Profiles

**DOI:** 10.3390/ijerph18147431

**Published:** 2021-07-12

**Authors:** Gabriela Martins Costa Gomes, Wilfried Karmaus, Vanessa E. Murphy, Peter G. Gibson, Elizabeth Percival, Philip M. Hansbro, Malcolm R. Starkey, Joerg Mattes, Adam M. Collison

**Affiliations:** 1 Priority Research Centre GrowUpWell®, Hunter Medical Research Institute, The University of Newcastle, Newcastle, NSW 2308, Australia; gabriela.martinscostagomes@uon.edu.au (G.M.C.G.); vanessa.murphy@newcastle.edu.au (V.E.M.); elizabeth.percival@uon.edu.au (E.P.); joerg.mattes@newcastle.edu.au (J.M.); 2 School of Public Health, University of Memphis, Memphis, TN 38152, USA; karmaus1@memphis.edu; 3 Priority Research Centre for Healthy Lungs, Hunter Medical Research Institute, University of Newcastle, Newcastle, NSW 2308, Australia; peter.gibson@newcastle.edu.au (P.G.G.); philip.hansbro@newcastle.edu.au (P.M.H.); 4 Sleep Medicine Department, John Hunter Hospital, Newcastle, NSW 2305, Australia; 5 Centre for Inflammation, Centenary Institute and University of Technology Sydney, School of Life Sciences, Faculty of Science, Sydney, NSW 2007, Australia; 6 Department of Immunology and Pathology, Central Clinical School, Monash University, Melbourne, VIC 3800, Australia; malcolm.starkey@monash.edu; 7 Paediatric Respiratory & Sleep Medicine Department, John Hunter Children’s Hospital, Newcastle, NSW 2305, Australia

**Keywords:** air pollutants, cord blood, asthma, prenatal risk factors, particulate matter

## Abstract

Air pollution exposure during pregnancy may be a risk factor for altered immune maturation in the offspring. We investigated the association between ambient air pollutants during pregnancy and cell populations in cord blood from babies born to mothers with asthma enrolled in the Breathing for Life Trial. For each patient (*n* = 91), daily mean ambient air pollutant levels were extracted during their entire pregnancy for sulfur dioxide (SO_2_), nitric oxide, nitrogen dioxide, carbon monoxide, ozone, particulate matter <10 μm (PM_10_) or <2.5 μm (PM_2.5_), humidity, and temperature. Ninety-one cord blood samples were collected, stained, and assessed using fluorescence-activated cell sorting (FACS). Principal Component (PC) analyses of both air pollutants and cell types with linear regression were employed to define associations. Considering risk factors and correlations between PCs, only one PC from air pollutants and two from cell types were statistically significant. PCs from air pollutants were characterized by higher PM_2.5_ and lower SO_2_ levels. PCs from cell types were characterized by high numbers of CD8 T cells, low numbers of CD4 T cells, and by high numbers of plasmacytoid dendritic cells (pDC) and low numbers of myeloid DCs (mDCs). PM_2.5_ levels during pregnancy were significantly associated with high numbers of pDCs (*p* = 0.006), and SO_2_ with high numbers of CD8 T cells (*p* = 0.002) and low numbers of CD4 T cells (*p* = 0.011) and mDCs (*p* = 4.43 × 10^−6^) in cord blood. These data suggest that ambient SO_2_ and PM_2.5_ exposure are associated with shifts in cord blood cell types that are known to play significant roles in inflammatory respiratory disease in childhood.

## 1. Introduction

Asthma is the most common medical condition during pregnancy, with up to 45% of pregnant women with asthma requiring medical care for it [1,2]. Maternal asthma has been found to increase the risk of adverse neonatal outcomes, including respiratory complications [3,4,5], and it has been reported that prenatal exposure to air pollutants such as particulate matter, ozone, and nitrogen oxides increased the risk of transient tachypnea of the newborn, asphyxia, and respiratory distress syndrome [6].

Air pollution refers to the mixture of gases and particulate matter (PM) composed of organic chemicals, metals, gases, biological agents, volatile organic compounds, and minerals carried in the air [7,8,9], which collectively have been linked to harm in nearly every organ in the body [10,11]. Around 91% of the world’s population lives in areas where the levels of air pollutants exceed the World Health Organization (WHO) safe limits [12]. The primary gaseous contaminants are carbon monoxide (CO) and dioxide (CO_2_), nitrogen dioxide (NO_2_), ozone (O_3_), and sulfur dioxide (SO_2_) [13]. According to the WHO, it is estimated that environmental exposure to PM is responsible for significant morbidity and mortality, including ~16% of lung cancer and 11% of chronic obstructive lung disease (COPD) deaths, and more than 20% of ischemic heart disease and stroke [12].

There is a growing body of epidemiological evidence that links exposure to outdoor air pollution and worsening of pre-existing asthma. These changes have been linked to enhanced inflammation through both innate and adaptive immune pathways as reviewed by Bontinck et al. [14]. Such immunological shifts will alter the interaction between the maternal and developing infant immune systems, and in line with the developmental origins of disease hypothesis, have the potential to shape the future susceptibility to disease [15]. The placenta is a natural barrier between mother and fetus during pregnancy; however, it is not an impenetrable barrier, and environmental air pollutants can cross the placenta; therefore, recent studies have explored the impact of air pollutant exposure during gestation on the unborn child [16,17]. Environmental air pollutants particles that translocate into and cross the placental barrier may also indirectly impact the developing immune system of the child through altering the maternal immune environment [16,17]. Maternal exposure to deleterious environmental factors may negatively impact the developing fetus either directly or indirectly and has been shown to have specific effect on birth weight [18,19,20], potentially altering immune cell maturation and function, subsequently influencing the risk of postnatally acquiring inflammatory and allergic diseases [21].

Neonatal susceptibility to environmental pollutants may be due to either direct or indirect effects on various cell types that exert influence over key processes, including cell differentiation, proliferation, and/or maturation [22]. Previous studies have demonstrated that environmental factors including NO_2_, SO_2_, PM < 10 μm in diameter (PM_10_), PM < 2.5 μm (PM_2.5_), and polycyclic aromatic hydrocarbons (PAHs) are associated with different cell populations measured in cord blood [23,24,25,26,27]. For instance, global cord blood lymphocyte levels and activity are associated with prenatal air pollution exposure to PAHs and PM_2.5_ [23,28,29]. Thus, early exposure to air pollution and environmental contaminants may affect the newborn immune system [30]. As many of these changes persist throughout life, alterations in the development of the immune system may be linked to an increased risk of an allergic phenotype in childhood and beyond [31,32,33].

Although there are reports of the effects of environmental factors on cord blood cell populations, previous studies did not phenotype the cell populations in great detail [23,24,25,26,27,28,29,34] and did not account for the influence that maternal asthma has on the newborn immune system [35]. Thus, we investigated the association between levels of ambient air pollutants during pregnancy (SO_2_, nitric oxide (NO), NO_2_, CO, O_3_, PM_10_, and PM_2.5_) on populations of well-defined cord blood cells using exposure dimensionality-reduction methods (principal component analysis, PCA) and linear regression approaches to assess associations between maternal exposure and cord blood cell populations from babies born to asthmatic mothers.

## 2. Materials and Methods

### 2.1. Study Design and Participants

Pregnant asthmatic women, 18 years or older, with physician-diagnosed asthma, were enrolled in the Breathing for Life Trial (BLT) [36]. The BLT is a multicenter (Brisbane (QLD), Canberra (ACT), Newcastle (NSW), and Sydney (NSW)) randomized controlled trial of asthma management during pregnancy, with follow-up into childhood. Maternal drug or alcohol dependence, chronic oral corticosteroid use, chronic lung disease other than asthma, concomitant chronic illness were exclusion criteria. Eligible mothers agreed to have an interviewer-administered questionnaire conducted during enrolment and information ascertained covered sociodemographic characteristics and lifestyle factors. Participants self-reported age, ethnicity, parity, health status, drug/alcohol dependence. Height and weight were also measured during the first visit. Enrolled mothers at the Newcastle site who consented to participate in the infant follow-up had cord blood collected after delivery. Trained staff extracted information from medical records on gestational age at birth, birth weight, birth length, mode of delivery, maternal and neonatal complications. In this study, 91 pregnant asthmatic woman and their babies were included from mothers previously enrolled in the Breathing for Life Trial (Figure S1).

### 2.2. Ethics Statement

The study was approved by the Hunter New England Human Research Ethics Committee (Ref no 12/10/17/3.04) and all women provided written informed consent before participation.

### 2.3. Cord Blood Collection

Cord blood samples were collected at John Hunter Hospital (New Lambton Heights, NSW, Australia) immediately after birth (*n* = 91) by needle puncture of the umbilical vein after the umbilical cord was detached from the infant. All samples were transferred into EDTA tubes to be processed by trained staff within six hours.

### 2.4. Flow Cytometry Analysis

Cord blood cells were stained in whole blood and subsets were pre-defined based on specific surface markers as follows: Eosinophils (CD45^+^, CD193^+^, CD16^−^), neutrophils (CD45^+^, CD193^−^, CD16^+^), CD4 T lymphocytes (CD3^+^, αβ T-cell receptor [TCR]^+^, CD4^+^), CD8 T lymphocytes (CD3^+^, αβTCR^+^, CD8^+^), regulatory T (Treg) cells (CD3^+^, αβTCR^+^, CD4^+^, CD25^+^, CD127^−^), natural killer (NK) cells (CD14^−^, CD3^−^, CD56^+^, CD16^+^), myeloid dendritic cells (mDCs—CD3^−^, CD19^−^, CD56^−^, CD14^−^, HLA-DR^+^, CD303^−^, CD16^+/−^, CD1c^+/−^, CD141^+^), plasmacytoid dendritic cells (pDCs—CD3^−^, CD19^−^, CD56^−^, CD14^−^, HLA-DR^+^, CD303^+^), innate lymphoid cells (ILCs) type 1 (ILC1—CD45^+^, lineage-negative (Lin^–^; CD3, TCR-αβ, TCR-γδ, CD19, CD11c, CD94, CD14, CD1a, CD34, CD123, CD303, FcεRIα), CD127^+^, CD161^+^, CD117^–^, CRTh2^–^, NKp44^–^), ILCs type 2 (ILC2—CD45^+^, Lin^–^, CD127^+^, CD161^+^, CRTh2^high^, CD117^–^), ILCs type 3 (ILC3) natural-cytotoxicity-receptor-negative (NCR^–^; CD45^+^, Lin^–^, CD127^+^, CD161^+^, CD294^–^, CD117^+^, NKp44^–^), ILC3 natural-cytotoxicity-receptor-positive (NCR^+^; CD45^+^, Lin^–^, CD127^+^, CD161^+^, CD294^−^, CD117^+^, NKp44^+^) (Table S1). After 30 min of incubation, red blood cells were lysed, and cells were fixed using BD FACS™ Lysing Solution and washed. Samples were stored at 4 °C and acquired within 48 h on a LSRFortessa X-20 flow cytometer (BD Biosciences, San Diego, CA, USA). For the granulocyte panel, NK, lymphocytes and DCs panels a total of 1,000,000 events were acquired and recorded for each subject. The ILCs panels had a total of 2,500,000 events recorded for each subject. Analyses of cell types were conducted with FlowJo software (v 10.5, Flow Jo LLC, Ashland, OR, USA). Results are shown as positive cells in 10^3^ of CD45 positive cells (for granulocytes, ILCs), as positive cells in 10^3^ of CD3 positive cells (NK cells, lymphocytes), and as positive cells in 10^3^ of HLA-DR (DCs).

### 2.5. Air Pollutant Assessment

Maternal exposure to air pollutants throughout pregnancy was approximated using data from the New South Wales (NSW) Air Quality Monitoring Network [37]. NSW air quality monitoring is achieved through an extensive network of National Association of Testing Authorities-accredited air quality monitoring stations. It reports the data as ambient concentrations and air quality index values, which are stored in a searchable public database. The data undergo rigorous quality assurance processes to ensure reliability. Quality assurance procedures are implemented, both in-the-field and post-data-collection, to ensure that air quality and meteorological parameters measured by the Office of Environment and Heritage air quality monitoring network are reliable. Data are available for the duration of the study period 2017–2019. All air pollution monitors used within this study contribute data on a regular basis and data are updated every morning [37,38].

Air pollutant levels were extracted as the mean daily level across pregnancy from hourly measurements. Levels were obtained for SO_2_, NO, NO_2_, CO, O_3_, PM_10_, PM_2.5_, humidity, and temperature. For each air pollutant, the trimesters’ mean level throughout pregnancy was normalized as a quotient of the hourly temperature and humidity at the time of collection prior to further analysis.

**Figure 1:**
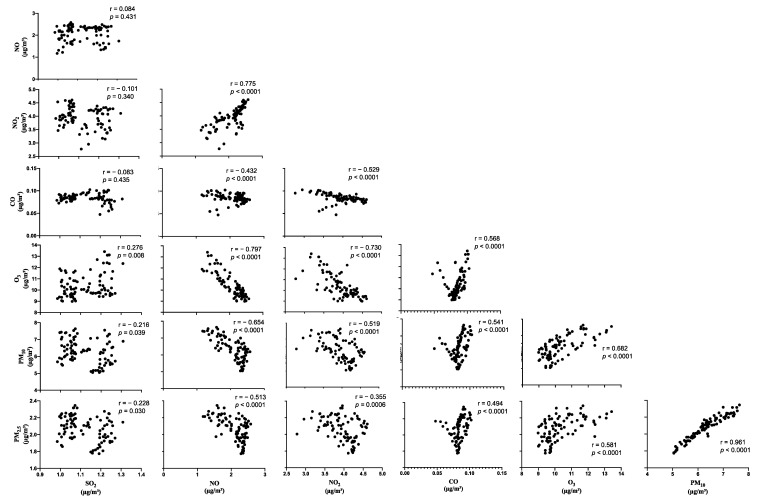
Correlation plots between SO_2_, NO, NO_2_, CO, O_3_, PM_10_, and PM_2.5._ Air pollutant levels are represented as the mean daily level across pregnancy from hourly measurements. Each air pollutant was normalized as a quotient of the hourly temperature and humidity at the time of collection.

Air pollution exposure data during pregnancy and the distribution of prenatal risk factors are shown in Table 1. The relationship among the pollutants is shown in Figure 1.

### 2.6. Statistical Analysis

Analyses were performed using Stata IC 16.1 (Stata Corporation, College Station, TX, USA). Prior to the analysis of individual air pollutants and cord blood cells, to avoid multicollinearity, PCs of the seven air pollutants were identified. A similar approach was conducted to capture latent features or correlated cells. Through this multivariate analysis, it was possible to select subgroups of pollutants and cells that comprised multiple correlated individual markers and explain their variance [39]. The PCA approach first linearly re-arranged the original correlated variables into fewer new integrated variables (PCs). After varimax rotation, the PCA reduced the data dimensionality and increased interpretability and minimized information loss creating new uncorrelated variables that successively maximized the variance of the PC capturing the seven air pollutants. Hence, PCA found the most informative or explanatory features hidden in the data without a priori knowledge and reduced the number of tests needed for multiple pollutants.

PCA analysis was first applied for air pollutants (SO_2_, NO, NO_2_, CO, O_3_, PM_10_, PM_2.5_). This approach was also applied to cell types (eosinophils, neutrophils, Treg cells, CD4 T cells, CD8 T cells, NK cell, pDCs, mDCs, ILC1, CRTh2^high^ ILC2, NCR^−^ ILC3, NCR^+^ ILC3), focusing on factors with an eigenvalue of ≥1. Linear regression analyses were then applied to identify significant associations between the PCs of air pollutants and cell types. Here, PCs were considered for further analysis when *p* < 0.05. PCs previously selected were subsequently adjusted by potential confounders (other risk factors).

From the PCs considered for further analysis, individual components were selected based on PC loadings to further understand the specific air pollutants and cell types’ relationships. Here, PCs with a cut-off of ±0.4 were considered for further multivariable regression models. The following covariates of interest, based on work by Lura and collaborators [27], were included in the multiregression model: (a) male sex, (b) gestational age, (c) maternal smoking during pregnancy, (d) parity, (e) fetal heart rate decelerations during labor, and (f) mode of delivery. To adjust for multiple comparisons with Bonferroni correction, the error rate (0.05) was divided by the number of tests [40], which varied between the two multivariable regressions applied with the final cut off for significance in each analysis identified in the table legend.

## 3. Results

### 3.1. PCA from Air Pollutants Have Positive Loading for O_3_ and PM_2.5_

To condense the information of a large number of variables into a smaller set of new composite dimensions, with a minimum loss of information, PCAs were applied to air pollutants and cell types separately. Two PCs were retained on the PCA for the analysis of inhaled air pollutants during pregnancy. These two PCs account for 75.0% of the variance in the original seven variables. The first PC is described by having positive loading for O_3_ and the second had higher loadings for PM_2.5_ (Table 2, Figure S2).

### 3.2. PCA from Cell Types Have Positive Loading for CD8 T cells, ILC1, CRTh2^high^ ILC2, NCR^−^ ILC3, Neutrophils, pDCs, and NCR^+^ ILC3

For the analysis of cord blood cell types, four PCs were retained in the PCA, which together accounted for 60.7% of the variation in the original 12 variables. PC1 presented high numbers of CD8 T cells, while PC2 had higher ILC1, CRTh2^high^ ILC2, and NCR^−^ ILC3 cell numbers. The third PC3 was loaded with neutrophils, and the fourth was characterized by pDCs and NCR^+^ ILC3 (Table 3, Figures S3 and S4).

### 3.3. Air Pollutant PC2 Associates with Cell Type PC1 and Cell Type PC4

To avoid multiple testing between all air pollutants and all cell types, the analysis first focused on associations between PCs of air pollutants and PCs of cell types. To this end, we conducted eight tests, not adjusting for potential confounders. These crude associations were considered for further regression analysis, if their *p*-value was <0.05 divided by the number of tests (*p* < 0.006); this resulted in three significant associations (Table 4).

After further adjustment for prenatal risk factors (male sex, gestational age, maternal smoking during pregnancy, parity, fetal heart rate decelerations during labor, and mode of delivery) in regression models, only associations between air pollutant PC2 and cell type PC1 (β = −0.429, CI = −0.618 to −0.239, *p* = 2.19 × 10^−5^); and air pollutant PC2 and cell type PC4 (β = 0.471, CI = 0.273 to 0.668, *p* = 8.91 × 10^−6^) maintained statistical significance (Table 5). Thus, only these two associations were considered for further analysis.

### 3.4. SO_2_ Associates with CD8 T Cells, CD4 T Cells and mDCs While PM_2.5_ Associates with pDCs in a Multipollutant Multivariable Regression Models

After screening for potential associations using PCs capturing air pollutants and a distinct PC capturing cell counts, the next steps focused on individual air pollutants and cell types identified in the associations between the PCs. The only PC from air pollutants associated with cell type PCs in the multivariable regression model was PC2 (Table 5). Thus, only PC2 from air pollutants was considered for the multipollutant multi-cell analyses (Table 2 and Table 5).

Regarding the two significant PC of the cells, CD4 T cells and CD8 T cells were considered for further analysis from PC1. For PC4 of the cells, pDC and NCR^+^ ILC3 had loadings >0.4, and mDCs presented loadings < −0.4 (Table 3 and Table 5, Figures S3 and S4). For this model, the following potential confounders were taken into consideration: male sex, gestational age, maternal smoking during pregnancy, parity, fetal heart rate decelerations during labor, and mode of delivery, with the addition of the concentration of the pollutants with the magnitude of 0.4 (PM_2.5_ and SO_2_).

In the second multipollutant multivariable regression model that considered pollutants selected in PC2 (air pollutants) and cells selected in PC1 (cell types), SO_2_ positively correlated with CD8 T cells (β = 260.242, CI = 99.087 to 421.397, *p* = 0.002), and negatively associated with CD4 T cells (β = −234.283, CI = −413.582 to −54.984, *p* = 0.011; Table 6). Here, applying the Bonferroni correction to four tests the critical *p*-value was 0.012.

Analyzing individual components of PC2 (air pollutants) and of PC4 (cell types), a positive association was found between PM_2.5_ and pDCs (β = 155.158, CI = 46.578 to 263.738, *p* = 0.006) and between SO_2_ and mDCs (β = 879.250, CI = 523.834 to 1234.666, *p* = 4.43 × 10^−06^). For this analysis with six tests, after Bonferroni correction, statistical significance was considered when *p* was less than 0.008 (Table 7).

## 4. Discussion

During pregnancy, the fetus has an intense and prolonged interaction with the mother at the maternal–fetus interface. Within this period, a complex network of interactions which provide passive immunity to the newborn, program the neonatal immune system, and tune its homeostatic regulation, is formed [35,41]. Maternal asthma represents a unique risk factor for childhood health, and asthmatic individuals are more susceptible to environmental exposures which can both interact with the maternal immune system and cross the placenta to directly interact with the developing child with detrimental health effects previously reported from early life onwards [16,17]. In this study, of the air pollutants investigated, only SO_2_ and PM_2.5_ were associated with differences in any of the 12 pre-defined cord blood cell populations after screening employing dimensionality-reduction. Mean daily local SO_2_ levels during gestation were negatively associated with CD4 T cells in cord blood, and positively associated with both CD8 T cells and mDCs numbers. In addition, mean daily local PM_2.5_ levels through pregnancy were positively associated with pDCs in the cord blood.

T-lymphocytes play an important role in the immune system, tailoring the body’s immune response to specific pathogens through the release of regulatory cytokines. T-cell development starts during the early weeks of gestation [32] during which toxic exposure can result in failure of stem cell formation or, in the later phases of gestation, can cause abnormal stem cell formation, interrupting cell migration and proliferation [42,43,44]. Based on cytokine production, activated CD4 T cells are predominantly classified as either T helper (Th) type 1 (Th1) lymphocytes that produce interferon (IFN)-γ, interleukin (IL)-2 and IL-12, or Th type 2 (Th2) lymphocytes that produce mainly IL-4, IL-5, and IL-13 [45]. The counter-regulation between Th1 and Th2 is capable of inhibiting or inducing the development of an allergic phenotype [46,47]. Exposure to immunotoxic compounds in utero can cause immuno-suppression and predispose to aberrant immune responses later in life [48].

Gaseous molecules can freely diffuse through biological membranes and SO_2_ inhalation can cause oxidative injury in the cardiovascular, respiratory, digestive, reproductive, endocrine, and neurological systems [49]. Several studies point to the relationship between SO_2_ exposure and inflammation; SO_2_ levels were significantly increased in acute pneumonia and chronic renal failure patients [50,51], and pro-inflammatory cytokines levels were increased in the lungs of mice after exposure to SO_2_ [52].

In this study, SO_2_ negatively associated with cord blood CD4 T cells, while positively associating with CD8 T cells. Air pollutant exposure during the early months of pregnancy has previously been shown to influence the Th1/Th2 homeostatic balance [23], and levels of cell proliferation in the cord blood have also been reported to be associated with air pollutant exposure ex vivo [53]. A recent experimental study showed epithelial damage and increased infiltration of inflammatory cells into the airways after PM_2.5_ exposure and also an immune imbalance of Th cells [54]. The exposure to PM_2.5_ also disturbs the balance of T helper 17 (Th17)/Treg cells [55]. Both acute and long-term exposure to high levels of PM_2.5_ were associated with alterations in differentially methylated regions of forkhead box P3 (Foxp3) [56].

Previous studies conducted in Australia report with similar levels of PM_2.5_ and identify an association between air pollutant exposure and pregnancy disorders such as hypertensive disorders during pregnancy and increased likelihood of gestational diabetes mellitus [57] which can further affect the fetus [58,59]. Another study conducted in the United States, also showing similar levels of PM_2.5_ in similar cohorts, has demonstrated that later phases of prenatal lung development may be particularly sensitive to the developmental toxicity of PM_2.5_ [60]. Although air pollution is a universal issue, it is likely that there are high-risk individuals who are susceptible to the greatest harm when exposed to PM_2.5_ [61]. It has further shown that PM_2.5_ can induce allergic airway inflammation [62], and trigger exacerbations in pre-existing asthma and COPD [63]. As smaller particles, PM_2.5_ is readily able to penetrate deeper into the lungs and cross into the bloodstream at a higher rate than larger particles [64], which can cause chronic inflammation in pregnant women and hamper fetal development [21,65,66]. Air pollution exposure during pregnancy has been linked to placental inflammation and impairment of placental function [67]. PM_2.5_ exposure may cause inflammatory responses in the placenta, which may be transmitted from the mother to the fetus and contribute to the development of abnormalities [68,69].

Interestingly, DC subsets quantified in the cord blood positively associated with environmental SO_2_ as well as PM_2.5_. DCs are a major link between the innate and the adaptive immune system. They recognize antigens through the expression of innate receptors such as toll-like receptor (TLR), and process and present fragments of these antigens on their cell surface to T-lymphocytes that then deliver effector responses. Studies show that a pro-inflammatory response in the airway mediated by TLR activation, might be stimulated by PM-associated biological components, such as pollen, bacteria, fungal spores, and viruses, as well as with soluble metals, and organic content [70,71,72,73,74]. Here, air pollutant measurements were taken in a region of Australia and included months with potentially increased air pollution due to environmental events such as bushfires which may also have contributed to the alterations seen in DC subsets [75,76,77,78].

PM acts on APCs, such as DCs, as an adjuvant. Cultured DCs stimulated by PM increase maturation with elevated expression of CD80 and major histocompatibility complex class II (MHC-II) and increased pro-inflammatory cytokine release [79,80]. PM-stimulation also promotes DC expression of C-C chemokine receptor type 7 (CCR7), which directs lymph-node homing [81] initiating the immune response cascade. Thus, enhanced DC maturation may promote an enhanced T-lymphocyte response to PM [79,82,83]. In individuals with existing asthma, increased pDC numbers and activity have been linked to acute exacerbations, particularly virally induced exacerbations [84]. Indeed, pDC function may play a significant role in the pathogenesis of asthma. We used animal models to demonstrate that pDCs deficient in TLR7 contribute to virally induced exacerbations of allergic airways disease that is reversed by TLR7 competent pDCs [85]. Thus, it is tempting to speculate that the increased pDC levels found in children whose mothers had highest exposure to PM_2.5_ may contribute to an increased susceptibility to develop virally induced wheeze and asthma later in life. This will be of interest as our cohort grows to an age where these measures can be evaluated. Children born to mothers with moderate to severe uncontrolled asthma during pregnancy are at increased risk of developing asthma and more commonly have lung function abnormalities [4,5]. However, studies have shown that with better asthma control during pregnancy and fewer exacerbations, there was a better respiratory health outcome for the children [86,87], suggesting that these pathways may be modified through improved maternal asthma management.

During pregnancy, maternal immune responses shift towards a type 2 (T2) predominance that promotes immunological tolerance towards the fetus. The balance of type 1 (T1) and T2 cytokines in pregnancy is thought to be crucial to maternal tolerance of the infant [88,89]. The fetal immune system is thought to be under the direct influence of the maternal immune response mounted at the fetus–maternal interface and studies have demonstrated that asthmatic individuals are susceptible to environmental exposure, and air pollution can cause exacerbations of pre-existing asthma [90,91,92]. Air pollution exposure during pregnancy was previously shown to be associated with reduced postnatal lung function [93], and it has been demonstrated that the effect of prenatal air pollution exposure on lung function at five weeks are sustained up to 11 years of age [94]. Perinatal air pollution exposure was also demonstrated to affect asthma onset during pre-school and school age periods [60,95]. It is emerging that there might be further life-long implications with several recent studies having shown that lung function in early life tracks into adulthood [96,97,98,99], and is associated with an increased risk of chronic respiratory diseases including asthma and COPD [100].

The findings observed in this study are limited to pregnancies in mothers with asthma, and the air pollution data available were generalized from local air pollution monitoring stations which are limited in their accuracy to the actual levels in the air breathed by the participants in their homes and workplaces. This study shares the limited sample size that is a common limitation of cord blood studies. Our sample included cord blood of 91 newborns, however, the advantages are the ability to access a suitable quantity of blood so early in life. This has enabled cord blood studies to make significant contributions to our understanding of early life immune and respiratory development [101,102,103,104,105,106]. In addition, all samples collected and analyzed in this study were from a population at high-risk of developing lung disease, who were infants born to asthmatic mothers, which may provide increased power to detect immune changes associated with subsequent lung disease.

In summary, higher levels of inhaled SO_2_ during pregnancy may have a direct effect, reducing cord blood CD4 T cells while increasing CD8 T cells and mDCs. It is suggested that inhaled PM_2.5_ exert their effects by increasing cord blood pDCs numbers. Through the evaluation of the association between ambient air pollutants during pregnancy and cord blood immune cell types, this study shows that SO_2_ and PM_2.5_ exposure during pregnancy are associated with shifts in cord blood cell types which may cause an inflammatory response in the placenta that may influence fetal development. Further follow-up studies of this cohort and complimentary mechanistic studies are required to elucidate if these immunological shifts persist into later life and/or are associated with increased risk of subsequent chronic disease.

## 5. Conclusions

In summary, higher levels of inhaled SO_2_ during pregnancy are associated with reduced cord blood CD4 T cells and increased CD8 T cells and mDCs. Local PM_2.5_ levels through pregnancy were also associated with increased cord blood pDCs numbers. Through the evaluation of the association between ambient air pollutants during pregnancies complicated by asthma and cord blood immune cell types, this study shows that SO_2_ and PM_2.5_ exposure is associated with shifts in cord blood cell types that are known to play significant roles in inflammatory respiratory disease in childhood. Further follow-up studies of this cohort and complimentary mechanistic studies are required to elucidate if these immunological shifts persist into later life and/or are associated with subsequent chronic disease.

## Figures and Tables

**Table 1:** Population characteristics.

	Mean (Min–Max)/*n* (%)	Total (*n*)
**Demographic Characteristics**		
Maternal smoking during pregnancy	11 (12.1)	91
Maternal recurrent asthma exacerbation during pregnancy	4 (4.9)	81
Maternal age at delivery	30.0 (19.0–41.5)	91
Gestational age at delivery (weeks)	39 (34–41)	91
Caesarean section	33 (36.3)	90
Mode of labor, spontaneous	16 (17.8)	90
Mode of labor, augmented	9 (10.0)	90
Mode of labor, induced	48 (53.3)	90
Fetal heart rate decelerations during labor	29 (32.2)	90
Male sex	49 (53.8)	91
Older siblings	46 (51.1)	90
Birth weight (kg)	3.5 (2.1–4.9)	90
Birth length (cm)	51.6 (30.7–58.0)	86
**Air pollution exposure during pregnancy—** **Mean daily concentration**		
SO_2_ (µg/m³)	4.2 (3.5–5.0)	91
PM_10_ (µg/m³)	23.6 (20.4–26.8)	91
PM_2.5_ (µg/m³)	7.8 (7.1–8.5)	91
NO (µg/m³)	7.9 (4.1–9.7)	91
NO_2_ (µg/m³)	15.0 (10.2–17.6)	91
CO (µg/m³)	0.3 (0.2–0.4)	91
O_3_ (µg/m³)	39.0 (34.6–44.4)	91
Humidity (%)	69.9 (66.1–73.9)	91
Temperature (^°^C)	18.6 (16.8–20.7)	91

SO_2_ sulfur dioxide, PM_10_ particulate matter < 10 µm in diameter, PM_2.5_ particulate matter < 2.5 µm in diameter, NO nitric oxide, NO_2_ nitrogen dioxide, CO carbon monoxide, O_3_ ozone.

**Table 2:** Components loadings for air pollutant PC1 and PC2 after varimax rotation.

Air Pollutants	PC1	PC2
SO_2_	0.144	−0.719
NO	−0.464	−0.002
NO_2_	−0.430	0.260
CO	0.268	0.051
O_3_	0.505	−0.197
PM_10_	0.396	0.386
PM_2.5_	0.309	0.474

SO_2_ sulfur dioxide, PM_10_ particulate matter < 10 µm in diameter, PM_2.5_ particulate matter < 2.5 µm in diameter, NO nitric oxide, NO_2_ nitrogen dioxide, CO carbon monoxide, O_3_ ozone.

**Table 3:** Component loadings for cell types PC1, PC2, PC3, and PC4 after varimax rotation.

Cell Types	PC1	PC2	PC3	PC4
Eosinophils	0.006	0.045	−0.683	−0.005
Neutrophils	−0.002	0.051	0.698	−0.014
Treg	−0.325	−0.125	0.036	0.304
TCD4	−0.583	−0.018	−0.018	−0.163
TCD8	0.557	0.058	−0.055	−0.003
NK cells	0.362	−0.234	0.110	−0.050
pDC	0.183	−0.064	−0.063	0.585
mDC	0.196	0.047	0.071	−0.402
ILC1	−0.172	0.488	0.041	0.064
CRTh2^high^ ILC2	0.063	0.564	−0.093	−0.158
NCR^−^ ILC3	0.086	0.586	0.078	0.115
NCR^+^ ILC3	0.012	0.123	0.065	0.577

**Table 4:** Crude regression model of PCs of air pollutants and cord blood cell types. After Bonferroni correction, significance was considered when *p* < 0.006.

Crude Regression	PC1 Cell Types		PC2 Cell Types		PC3 Cell Types		PC4 Cell Types	
	Coef. (95% CI)	*p* Value	Coef. (95% CI)	*p* Value	Coef. (95% CI)	*p* Value	Coef. (95% CI)	*p* Value
PC1 Air pollutant	−0.021 (−0.173; 0.129)	0.776	0.018 (−0.129; 0.165)	0.804	−0.055 (−0.198; 0.087)	0.443	0.083 (−0.053; 0.219)	0.230
PC2 Air pollutant	−0.411 (−0.632; −0.190)	0.0004	−0.187 (−0.415; 0.040)	0.106	−0.320 (−0.535; −0.106)	0.004	0.495 (0.305; 0.684)	1.29 × 10^−6^

**Table 5:** Multivariable regression model of PCs of air pollutants previously associated with PCs of cord blood cell types in an univariable regression model. After Bonferroni correction, significance was considered when *p* < 0.016.

Multivariable Regression *	PC1 Cell Types		PC3 Cell Types		PC4 Cell Types	
	Coef. (95% CI)	*p* Value	Coef. (95% CI)	*p* Value	Coef. (95% CI)	*p* Value
PC2 Air pollutant	−0.429 (−0.618; −0.239)	2.19 × 10^−5^	−0.192 (−0.390; 0.005)	0.056	0.471 (0.273; 0.668)	8.91 × 10^−6^

Coef. coefficient; CI confidence interval. * Adjusted for male sex, gestational age, maternal smoking during pregnancy, parity, fetal heart rate deceleration during labor, mode of delivery.

**Table 6:** Multipollutant multivariable regression models of pollutants and cord blood cells previously associated in PCA. PC2 from air pollutants and PC1 from cell types with ≥0.4 for the highest loadings and ≤−0.4 for the lowest loadings. After Bonferroni correction, significance was considered when *p* < 0.012.

	Multipollutant Multivariable Model *			
	PM_2.5_		SO_2_	
	Coef. (95% CI)	*p* Value	Coef. (95% CI)	*p* Value
TCD8 ^†^	−4.268 (−104.138; 95.602)	0.932	260.242 (99.087; 421.397)	0.002
TCD4 ^†^	63.128 (−47.986; 174.242)	0.262	−234.283 (−413.582–54.984)	0.011

PM_2.5_ particulate matter < 2.5 µm in diameter; SO_2_ sulfur dioxide; Coef coefficient; CI confidence interval; * Adjusted for male sex, gestational age, maternal smoking during pregnancy, parity, fetal heart rate deceleration during labor, mode of delivery, SO_2_, and PM_2.5_. ^†^ Results are expressed in 10^3^ of CD3 positive cells.

**Table 7:** Multipollutant multivariable regression models of cord blood cells and pollutants previously associated in PCA. PC2 from air pollutants and PC4 from cell types with ≥0.4 for the highest loadings and ≤−0.4 for the lowest loadings. After Bonferroni correction, significance was considered when *p* < 0.008.

	Multipollutant Multivariable Model *			
	PM_2.5_		SO_2_	
	Coef. (95% CI)	*p* Value	Coef. (95% CI)	*p* Value
pDC ^†^	155.158 (46.578; 263.738)	0.006	−169.378 (−344.588; 5.832)	0.058
mDC ^†^	−90.800 (−311.056, 129.456)	0.414	879.250 (523.834; 1234.666)	4.43 × 10^−6^
NCR ^+^ ILC3 ^‡^	0.149 (0.006; 0.290)	0.041	0.011 (−0.219; 0.240)	0.927

PM_2.5_ particulate matter < 2.5 µm in diameter; PM_10_ particulate matter < 10 µm in diameter; SO_2_ sulfur dioxide; Coef coefficient; CI confidence interval; * Adjusted for male sex, gestational age, maternal smoking during pregnancy, parity, fetal heart rate deceleration during labor, mode of delivery, SO_2_, and PM_2.5_. ^†^ Results are expressed in 10^3^ of HLA-DR positive cells. ^‡^ Results are expressed in 10^3^ of CD45 positive cells.

## Data Availability

The data presented in this study is available upon request to the corresponding author.

## Supplementary Materials

The following are available online at https://www.mdpi.com/article/10.3390/ijerph18147431/s1, Table S1: Antibodies used in flow cytometry analysis, Figure S1: Flow chart. Recruitment, collection of cord blood samples and maternal air pollutant exposure assessment. FACS, fluorescence-activated cell sorting, Figure S2: Component loadings (A) and score variables (B) from the PCA (air pollutant) analyses, Figure S3: Component loadings from the PCA (cell type) analyses, Figure S4: Score variables from the PCA (cell type) analyses.
