# Supplementary material for: Environmental Air Pollutants Inhaled during Pregnancy Are Associated with Altered Cord Blood Immune Cell Profiles
Source: Int J Environ Res Public Health. 2021 Jul 12;18(14):7431. doi: 10.3390/ijerph18147431 (PMC8303567; doi:10.3390/ijerph18147431)
Supplement: Supplementary file 1 [file ijerph-18-07431-s001.zip › ijerph-1252090-supplementary.pdf]

Article

Environmental Air Pollutants Inhaled During Pregnancy are Associated with Altered Cord Blood Immune Cell Profiles

Gabriela Martins Costa Gomes [1], Wilfried Karmaus [2], Vanessa E. Murphy [1], Peter G. Gibson [3,4], Elizabeth Percival [1], Philip M. Hansbro [3,5,6], Malcolm R Starkey [7], Joerg Mattes [1,8], Adam M. Collison [1,*]

[1] Priority Research Centre GrowUpWell®, Hunter Medical Research Institute, The University of Newcastle, Newcastle, NSW 2308, Australia; gabriela.martinscostagomes@uon.edu.au (G.M.C.G.); vanessa.murphy@newcastle.edu.au (V.E.M.); elizabeth.percival@uon.edu.au (E.P.); joerg.mattes@newcastle.edu.au (J.M.)

[2] School of Public Health, University of Memphis, Memphis, TN 38152, USA; karmaus1@memphis.edu (W.K.)

[3] Priority Research Centre for Healthy Lungs, Hunter Medical Research Institute, University of Newcastle, Newcastle, NSW 2308, Australia; peter.gibson@newcastle.edu.au (P.G.G.); philip.hansbro@newcastle.edu.au (P.M.H.)

[4] Sleep Medicine Department, John Hunter Hospital, Newcastle, NSW 2305, Australia; peter.gibson@newcastle.edu.au (P.G.G.)

[5] Centre for Inflammation, Centenary Institute, Sydney, NSW 2025, Australia; philip.hansbro@newcastle.edu.au (P.M.H)

[6] Faculty of Science, School of Life Sciences, University of Technology Sydney, Sydney, NSW 2050, Australia

[7] Department of Immunology and Pathology, Central Clinical School, Monash University, Melbourne, 3004, Australia; malcolm.starkey@monash.edu (M.R.S.)

[8] Paediatric Respiratory & Sleep Medicine Department, John Hunter Children's Hospital, Newcastle, NSW 2305, Australia

*Correspondence: adam.collison@newcastle.edu.au; Tel.: +61-2-4042-0219

Online Supplement Data:

Table S1. Antibodies used in flow cytometry analysis.

CD Antigen	Other Names	Isotope	Clone	Company	Brief Description
**CD1a**	HTA1	PE	HI149	BD bioscience	Lipid Antigen presentation
**CD1c**	BDCA-1	BB515	F10/21A3	BD bioscience	Type 1 transmembrane glycoprotein
**CD3**	T3	BV510	UCHT1	BD bioscience	TCR co-receptor (Tcell marker)
**CD3**	T3	PE-Cy7	SK7	BD bioscience	TCR co-receptor (Tcell marker)
**CD4**	T4	APC	RPA-T4	BD bioscience	TCR co-receptor (Tcell marker)
**CD8**	T8	APC-H7	SK1	BD bioscience	TCR co-receptor (Tcell marker)
**CD11c**	p150	PE	B-ly6	BD bioscience	Adhesion
**CD14**	LPS R	PE	M5E2	BD bioscience	R for complex of LPS and LBP, innate immune response
**CD14**	LPS R	PerCP Cy5.5	M5E2	BD bioscience	R for complex of LPS and LBP, innate immune response
**CD16**	FcγRIIIA	BV421	3G8	BD bioscience	Low affinity Fcγ receptor, mediates phagocytosis and ADCC, degranulation
**CD19**	B4	PE	HIB19	BD bioscience	BCR Coreceptor, signalling
**CD19**	B4	PE-Cy7	SJ25C1	BD bioscience	BCR Coreceptor, signalling
**CD25**	IL-2Rα	PE	M-A251	BD bioscience	IL-2 receptor α chain
**CD34**	gp105-120	PE	581	BD bioscience	Adhesion
**CD45**	LCA	APC	HI30	BD bioscience	Activation, signalling
**CD45**	LCA	APC Cy7	2D1	BD bioscience	Activation, signalling
**CD56**	NCAM1	PE-Cy7	B159	BD bioscience	Glycosylated adhesion protein
**CD94**	Kp43	PE	HP-3D9	BD bioscience	CD94/NKG2A inhibits NK function, CD94/NKG2C activates NK
**CD117**	cKit	PE-Cy7	104D2	BD bioscience	Signalling, crucial for HSC, gonadal and pigment stem cell growth and development
**CD123**	IL-3Rα	PE	9F5	BD bioscience	IL-3 receptor α chain
**CD127**	IL-7Rα	BV421	HIL-7R-M21	BD bioscience	IL-7 receptor α chain
**CD141**	BDCA-3	PE	1A4	BD bioscience	Thrombomodulin and fetomodulin
**CD161**	NKR-P1A	BV711	DX12	BD bioscience	NK cytotoxicity, induces immature thymocytes proliferation

**CD193**	CCR3	PE	5E8	BD bioscience	Leukocytes chemotaxis, HIV-1 coreceptor
**CD294**	CRTh2	BV786	BM16	BD bioscience	Regulates immune and inflammatory responses
**CD303**	BDCA-2	PE	201A	Biolegend	Type II transmembrane glycoprotein
**CD303**	BDCA-2	APC	201A	Biolegend	Type II transmembrane glycoprotein
**CD336**	NKp44	BB515	p44-8	BD bioscience	NK activation
**TCR-αβ**	TCR-αβ	PE	T10B9	BD bioscience	T cell receptor αβ chain
**TCR-αβ**	TCR-αβ	BV510	IP26	BD bioscience	T cell receptor αβ chain
**TCR γδ**	TCR γδ	PE	11F2	BD bioscience	T cell receptor γδ chain
**FcεRiα**	FcεRiα	PE	AER-37 (CRA-1)	Biolegend	High affinity IgE receptor
**HLA-DR**	MHC class II	BV510	G46-6	BD bioscience	Transmembrane heterodimeric glycoprotein

CD Cluster of differentiation, APC Allophycocyanin, APC Cy7/H7 Allophycocyanin cyanine 7, BB brilliant blue, BV brilliant violet, FITC Fluorescein isothiocyanate PE Phycoerythrin, PE Cy7 Phycoerythrin cyanine 7, PerCP Cy5.5 Peridinin-chlorophyll-protein cyanine 5.5

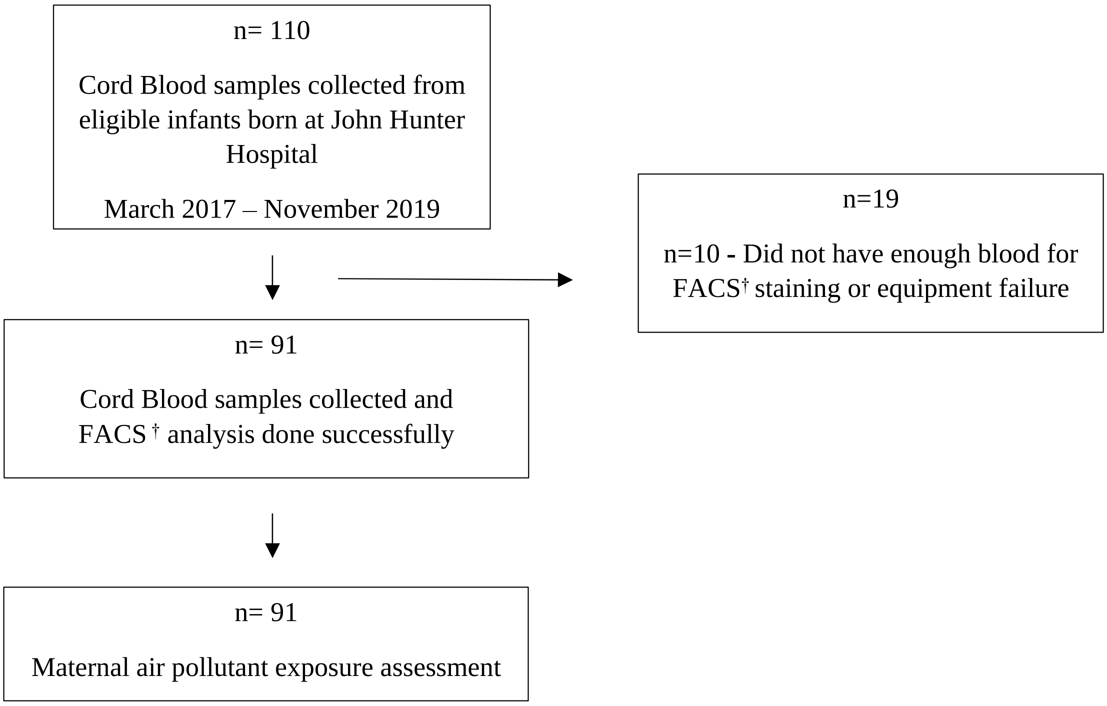

†FACS Fluorescence-activated cell sorting

Figure S1. Flow chart. Recruitment, collection of cord blood samples and maternal air pollutant exposure assessment. FACS, fluorescence-activated cell sorting.

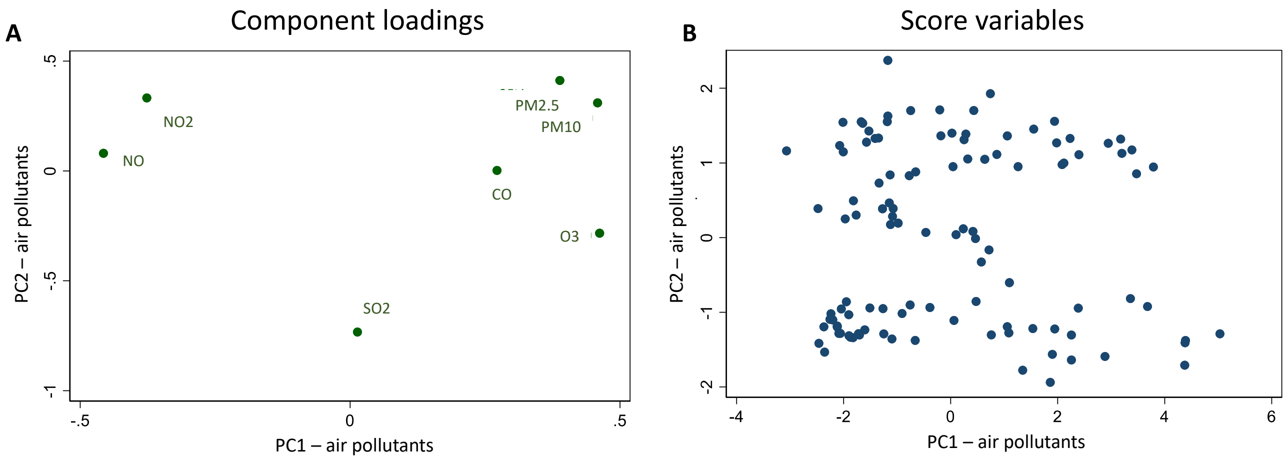

Figure S2. Component loadings (A) and score variables (B) from the PCA (air pollutant) analyses.

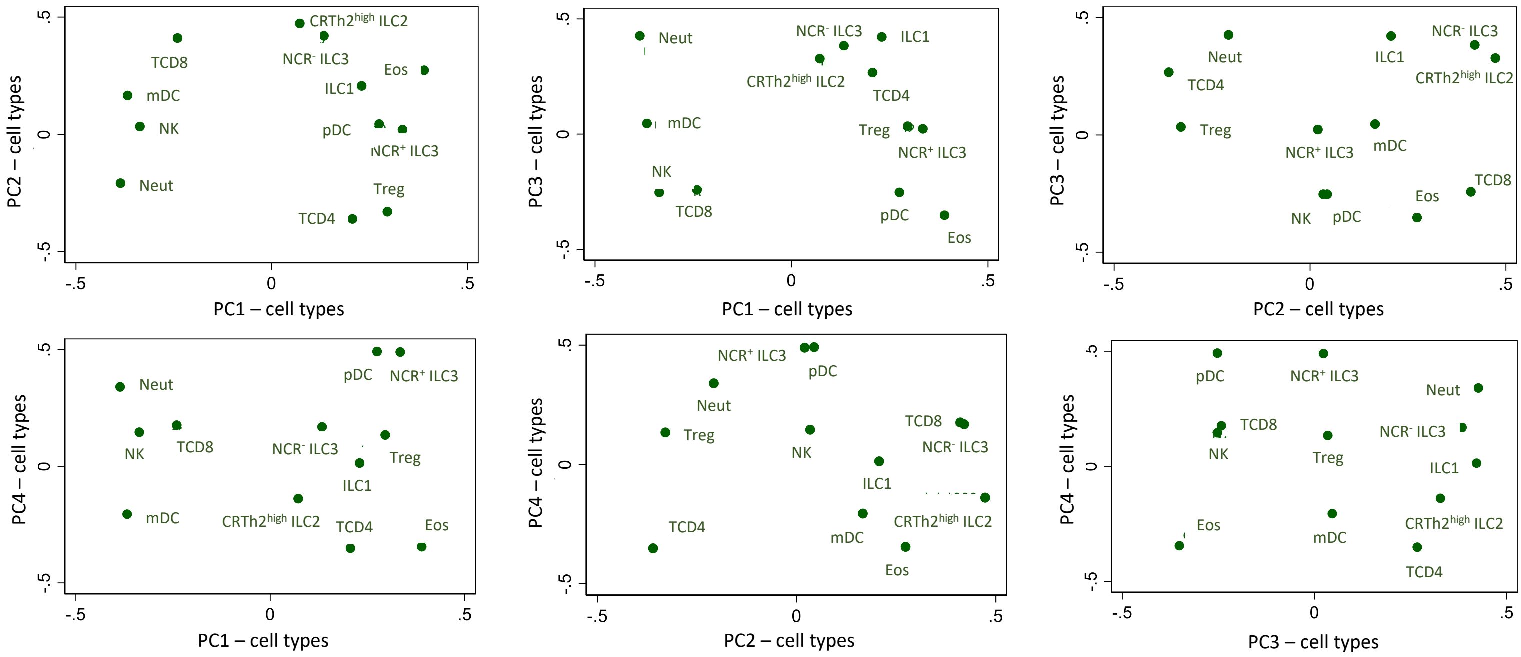

Figure S3. Component loadings from the PCA (cell type) analyses.

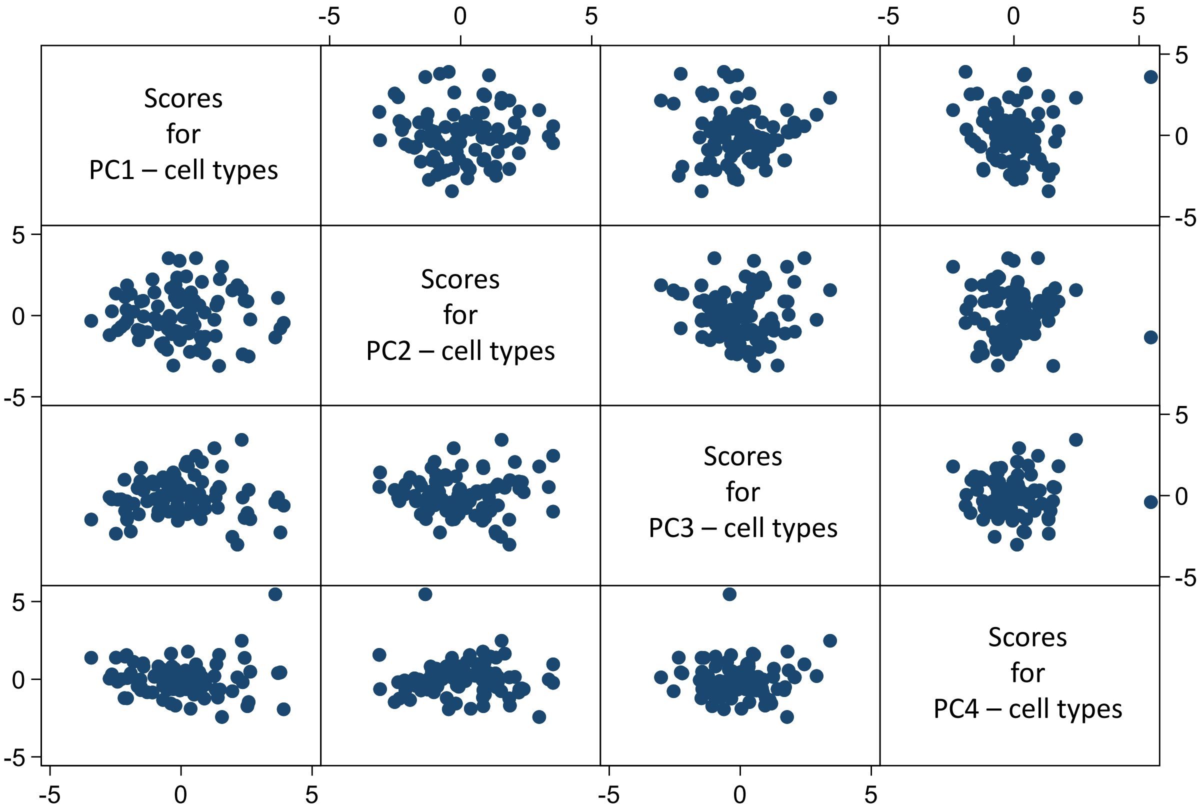

Figure S4. Score variables from the PCA (cell type) analyses.
